# COMPUTER-MEDIATED COMMUNICATION (CMC) AND SOCIAL ENGAGEMENT AMONG INDIVIDUALS WITH ALZHEIMER’S

**DOI:** 10.1093/geroni/igae098.0471

**Published:** 2024-12-31

**Authors:** Yun Leng Wong, Laura Hemmy, Mike Conway, Daniel Cabrera Lozoya, Kelvin Lim, Jude Mikal

**Affiliations:** University of Minnesota Twin Cities, Minnesota, United States; University of Minnesota, Minneapolis, Minnesota, United States; University of Melbourne, Melbourne, Victoria, Australia; University of Melbourne, Melbourne, Victoria, Australia; University of Minnesota, Minneapolis, Minnesota, United States; University of Minnesota, Minneapolis, Minnesota, United States

## Abstract

Normal aging confers a host of risk factors for loneliness and social isolation. Aging accompanied by Alzheimer’s-related changes in cognition and function may result in even higher risk of loneliness and social isolation. Nevertheless, older adults now represent the fastest-growing demographic of both Internet and social media users. Moreover, uptake continues to rise, with younger-older adults - or the Baby Boomer Generation - using social media on average two hours more per week than younger generations. This widespread increase in digital engagement has profound implications on loneliness and social isolation among individuals living with Alzheimer’s. In this study, we examine the lived experiences of individuals living with ADRD using blogs managed by individuals with early-onset AD to assess how the Internet enables them to stay connected and safe. Following a phenomenological approach, we apply rigorous bottom-up, constant-comparison methods to identify the impact of computer-mediated communication (CMC) technologies on social engagement, focusing on mental and physical health outcomes, social support exchange, and health resource sharing. Four key themes emerged from analysis: (1) the Internet as a gateway to formal networks of support and mentorship, (2) the ability to bridge geographical distances in pursuit of fitting and responsive support, (3) feelings of validation contribution coming from sharing experiences, and (4) feelings of embarrassment or forced awareness of functional and cognitive decline. Themes are contextualized, and used to make recommendations for the development of novel support exchange platforms focused on the needs of individuals with ADRD - and those with early-onset ADRD, specifically.

